# Classical hypercorrelation and wave-optics analogy of quantum superdense coding

**DOI:** 10.1038/srep18574

**Published:** 2015-12-22

**Authors:** Pengyun Li, Yifan Sun, Zhenwei Yang, Xinbing Song, Xiangdong Zhang

**Affiliations:** 1 School of Physics, Beijing Institute of Technology and Beijing Key Laboratory of Fractional Signals and Systems, 100081, Beijing, China

## Abstract

We report the first experimental realization of classical hypercorrelation, correlated simultaneously in every degree of freedom (DOF), from observing a Bell-type inequality violation in each DOF: polarization and orbital angular momentum (OAM). Based on such a classical hypercorrelation, we have realized the analogy of quantum superdense coding in classical optics. Comparing it with quantum superdense coding using pairs of photons simultaneously entangled in polarization and OAM, we find that it exhibits many advantages. It is not only very convenient to realize in classical optics, the attainable channel capacity in the experiment for such a superdense coding can also reach 3 bits, which is higher than that (2.8 bits) of usual quantum one. Our findings can not only give novel insight into quantum physics, they may also open a new field of applications in the classical optical information process.

Quantum entanglement plays a crucial role in various quantum information processing protocols, such as the one-way quantum computer^1^, quantum teleportation^2^, dense coding^3^, and some important quantum cryptographic schemes^4^. Paired photons that are entangled in multiple degrees of freedom (DOFs), which is called as hyperentanglement, have attracted a lot of interest in recent years^5^,^6^,^7^,^8^,^9^. It has been implemented in some real systems^10^,^11^,^12^,^13^,^14^,^15^. Due to the presence of quantum correlations in several DOFs, they offer significant advantages in quantum information processing, in particular in tasks such as superdense coding and multidimensional quantum cryptography^5^,^6^,^7^,^8^,^9^,^10^,^11^,^12^,^13^,^14^,^15^,^16^,^17^. For example, paired photons simultaneously entangled in polarization (spin) and orbital angular momentum (OAM) were shown to provide a channel capacity that exceeds the limit of standard quantum dense coding with linear optics^13^. It has been shown that the hyper-entangled state, involving two DOFs, can construct 16 Bell-like states^14^. Although only 7 from the group of 16 states can be distinguished, the attainable channel capacity in the experiment can reach 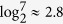 bits. The increase of capacity using hyperentanglement may present many potential applications for quantum communication protocols.

On the other hand, the violation of Bell’s inequality for the correlation among two different DOFs from the classical optical beam has been demonstrated experimentally^18^,^19^,^20^,^21^,^22^,^23^,^24^,^25^,^26^,^27^. Such a classical correlation is called “nonquantum entanglement” or “classical entanglement”^28^,^29^,^30^,^31^,^32^. Such a classical entanglement has been applied to resolve basic issues in polarization optics, simulate quantum walks, realize polarization metrology, implement analogy of quantum teleportation, perform quantum Fourier transformation and so on^33^,^34^,^35^,^36^,^37^,^38^,^39^. The problem is whether or not classical hypercorrelation defined as the correlation in several DOFs of the classical optical beams, which is analogy of quantum hyperentanglement, can be realized?

In this work, we present a method to construct classical hypercorrelation states, demonstrate their correlation properties from the Bell’s measurement used in tests of quantum non-locality. Based on these classical hypercorrelation states, we study the analogy of quantum superdense coding in classical optics.

## Results and Discussion

### Experimental demonstration of classical hypercorrelation

**Figure 1:**
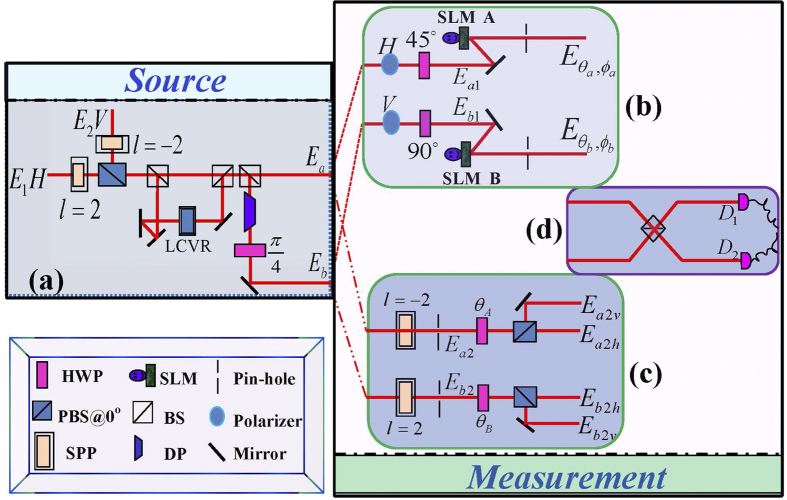
Experimental setup for the creation and analysis of classical hypercorrelation. (**a**) The preparation of classical hypercorrelation. 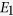 and 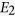 are two laser beams with the wavelength 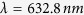. (**b**) and (**c**) show the demonstration of OAM correlation and polarization correlation, respectively. (**d**) Schematic picture for the measurement of the first-order field correlation. Legend of the main components (see also graphic symbol legend in the inset): SPP - spiral phase plant; PBS - polarizing beam-splitter; BS - nonpolarizing beam-splitter; LCVR - liquid crystal variable retarder; DP - dove prism; HWP - half-wave plate; P - polarizer; SLM A and SLM B - spatial light modulators; M - mirror; D_1_ and D_2_ - detectors for the intensity.

**Figure 2:**
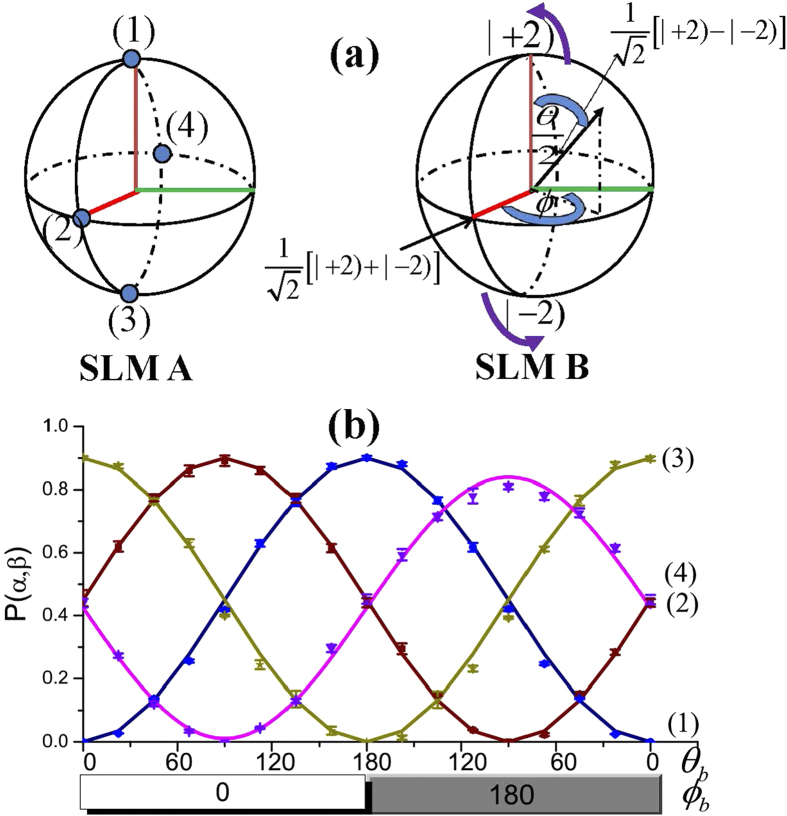
(**a**) Poincare sphere and Bloch sphere equivalent for 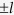 OAM states. (**b**) Bell curve for one great circle around the poles. Each curve corresponds to normalization correlation probabilities between a state around Bloch sphere on SLM B (upper-right inset) and one static state on SLM A (upper-left inset). Solid lines and dots represent the theoretical and experimental results, respectively.

The experimental setup we used to demonstrate hypercorrelation in classical optics is illustrated in Fig. 1. The scheme consists of two parts: the source generating classical hypercorrelation states (Fig. 1(a)), and the measurement insets for demonstrating the correlation properties (Fig. 2(b–d)). The source shown in Fig. 1(a) is constructed by two completely incoherent beams 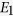 and 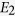, emitted from two independent 632.8 nm helium-neon (He-Ne) CW lasers. They are transformed into helical-wavefront laser beams with a spiral phase plate (SPP) which is designed to produce 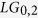-mode. In practice, our experimental setup indicates that the number of the OAM state is an arbitrary integer which is greater than zero. These light beams are then combined in a polarizing beam-splitter (PBS) that can separate horizontal 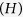 and vertical 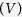 polarized light. The fields of two light beams satisfy complete incoherent condition 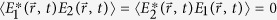. Here 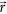 and 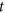 represent coordinates of space and time, respectively. The hybrid beam of 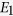 and 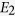 passes through the modified Mach-Zehnder interferometer (MZIM)^40^. The function of the reflector in one arm of the MZIM is to reverse the OAM of the beam from 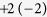 to 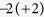. A liquid crystal variable retarder (LCVR) is introduced to ensure the incoherence of two beams. After the MZIM, the field becomes


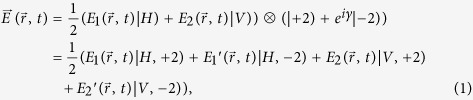


where the OAM, horizontal and vertical polarization components of the vortex beam are described by a slightly modified version of the familiar bra-ket notation of quantum mechanics^28^. The 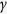 is a random phase and 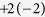 denotes the OAM eigenmode of order 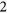 for the paraxial Laguerre-Gauss modes carrying 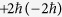 units. The symbol 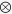 denotes the tensor product between the correlated states. Here 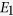, 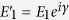, 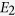, 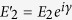 denote the fields behind the MZIM, and they satisfy complete incoherent conditions because the 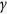 is a random phase. Subsequently, using a 50-50 nonpolarizing beam-splitter (BS), a dove prism (DP) and a half-wave plate (HWP) with fast axes at 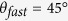, two new outputs can be written as


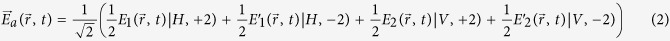


and


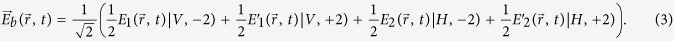


In order to analyze the hypercorrelation properties between 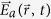 and 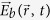, they will be divided into two parts. Using one of them to study the OAM correlation as shown in Fig. 1(b), the other part is used to study the polarization correlation as shown in Fig. 1(c). In Fig. 1(b), the beam from 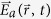 is filtered by a 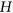 polarizer and passes through a HWP@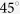, which can be expressed as


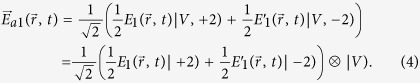


The beam from 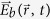 is filtered by a 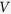 polarizer and passes through a HWP@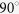, which can be expressed as


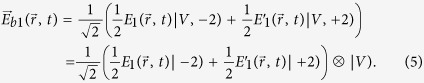


In order to analyze the OAM correlation properties between 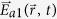 and 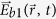, we take OAM basis similar to those in refs 41 and 42. A Bloch sphere is to be introduced to represent the first order transverse Laguerre-Gaussian modes as shown in Fig. 2(a). For a vortex beam in classical optics given order 2, 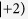 and 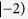, correspond to the north and south poles, respectively. In this case, each point on the Bloch sphere stands for a state, which can be described as


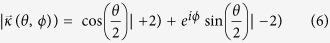


with 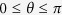 and 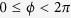. In our experiment, the Bloch vector 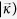 is acted as measurement basis, which corresponds to computer phase hologram carried by a spatial light modulator (SLM, Holoeye). With the help of a pin-hole (the function is filtering wave), the computer phase hologram can transform the target spatial mode into the pure Gaussian mode with 15% diffraction efficiency^43^. When the hybrid beams (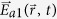 and 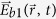) go through the mode splitters as shown in Fig. 1(b), the fields can be expressed in the following forms:


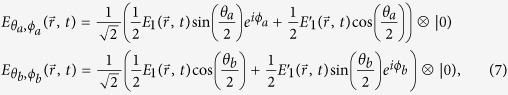


where 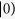 denotes the pure Gaussian mode. 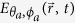 and 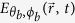 correspond to projection onto a state of the form of Eq. (6). The states can be selected in different directions, which are represented by the Bloch vectors 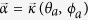 and 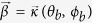 , respectively. In order to perform the Clauser–Horne–Shimony–Holt (CHSH) Bell’s measurement, we define the following correlation function^42^,^44^:


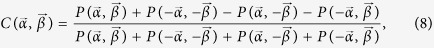


where 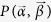 are normalized probabilities of states on the certain measurement basis for the OAM, which can be expressed as


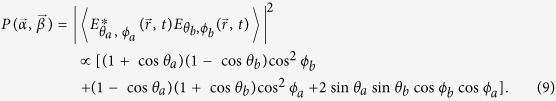


In the experiment, the correlation probabilities cannot be directly measured. They can be obtained through measuring the difference of light intensities at two export positions on Mach-Zehnder (MZ) interferometer as shown in Fig. 1(d), because 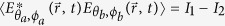. Here 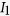 and 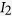 represent the light intensities at two export positions of the MZ interferometer. More information about the measurement method for the first-order field correlation has been given in ref. 27. Then, the CHSH measurement is


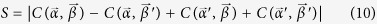


where 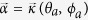, 
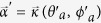
 and 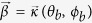, 
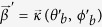
 correspond to two different measurement states, selected by the holograms on SLM A and SLM B, respectively.

Figure 2(b) shows experimental results for the normalized correlation probabilities as a function of 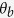 at various patterns on SLM B over the full range of possible value (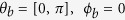 and 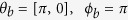) with fixing the angle of pattern on SLM A. The dots (circle, triangular, square and pentagram) and solid lines represent the experimental measurements and theoretical results, respectively. Here, the theoretical results are normalized by the experimental data. It can be seen that the experimental results are in good agreement with the theoretical calculations. As expected from Eq. (9), we observe sinusoidal fringes in the correlation probabilities. The fringe contrast is about 91.93%, which is much larger than 70.7%, as required for verification of Bell’s inequality^45^. The errors can be attributed mostly to misalignment of the interferometer.

The maximum value is obtained at 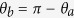 and 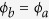, and the minimum value corresponds to the case for 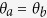 and 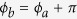. From Eq. (10) and experimental results in Fig. 2(a), Bell parameter S can be evaluated by selecting special angles 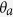, 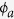, 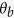 and 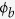. For example, when 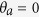, 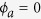, 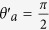, 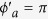, 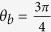, 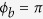, 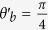 and 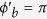, we obtain 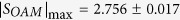, which yields the strongest violation of Bell’s inequalities in the OAM DOF.

Simultaneously, we can also test the polarization correlation using setup in Fig. 1(c). Here, the analysis of polarization is realized by erasing the distinguishing OAM labels. In Fig. 1(c), the combination of a SPP and a pin-hole, called mode splitter, is utilized to obtain pure Gaussian mode. The beams from 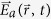 and 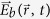 pass through the mode splitter, which can be expressed as


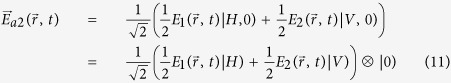


and


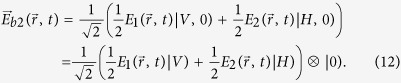


If a HWP and a PBS are introduced in each path, two polarized beams become then four beams. And the output light fields are described by 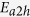, 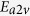, 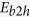 and 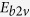, respectively, which can be modulated by varying the angle of HWPs (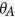 and 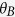). Subsequently, we perform the demonstration of classical polarization correlation. Similar to the method described in ref. 27, we can demonstrate that the maximum of Bell parameter reaches 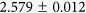 (see Section I in Supplemental Material for details).

Considering the above two aspects, we are sure that the classical hypercorrelation properties exist in polarization and OAM, which are similar to quantum correlation properties from hyper-entangled photon pairs. This means that the following Bell-like state can be produced in the measurement process of the first-order correlation^27^.


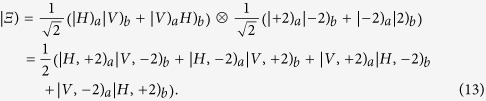


Here the normalizing conditions 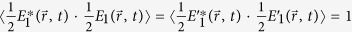 and 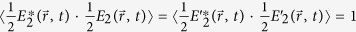 have been used. This is highly similar to the production of hyper-entangled photon pairs from spontaneous down-conversion of nonlinear crystal^14^, which means that the classical hyper-correlated state can be constructed. The problem is whether or not some unique phenomena such as superdense coding can be realized by applying such a classical hyper-correlated state, which is similar to the case in the quantum information process.

### Experimental realization of classical wave-optics analogy of quantum superdense coding

**Figure 3:**
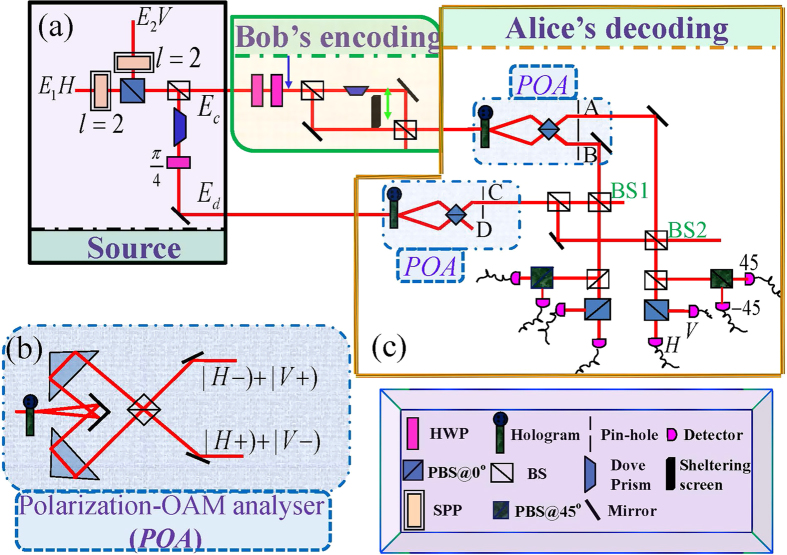
Experimental setup for superdense coding. The experiment consists of three distinct parts: (**a**) SOURCE: source; (**c**) BOB: Bob’s station, and ALICE: Alice’s analyzer. (**b**) Polarization-OAM analyser (POA). The incident light from the left is first split according to its 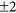-OAM content. Creating 0-OAM components are then combined on a PBS.

In order to study the classical analogy of quantum superdense coding, the experimental layout shown in Fig. 3 is considered. The experiment consists of three distinct parts: the hypercorrelation source generating nonlocal classical optical hypercorrelation states; Bob’s station for encoding the messages; finally, Alice’s analyzer to identify signals sent by Bob. Such an experimental scheme corresponds to that of the quantum superdense coding described in ref. 13.

Here the simplified form of the correlation source described in Fig. 1 has been used (see Fig. 3(a)), MZIM has been removed, the output fields in such a case are marked by 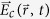 and 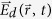:


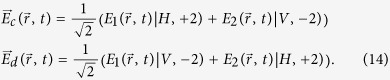


It is notable that the classical hypercorrelation that violates the Bell-type inequality can also be demonstrated in such a case (see Section II in Supplementary Material). In the following, we use such a hypercorrelation source to study the classical wave-optics analogy of quantum superdense coding. Similar to the scheme of quantum superdense coding by using hyperentanglement^13^,^14^, in our scheme encoding operations are also globally performed by manipulating separately the two DOFs of the beam 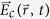, polarization and OAM. In Bob’s encoding station, Bob can encode his messages by using two HWPs in two channels where up or down channel can be only chosen for each operation. A DP is inserted in one of the two channels and HWPs are rotated at the correct angle 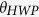 (see the Table 1 for operation details). Such operations transform 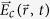 in Eq. (14) into


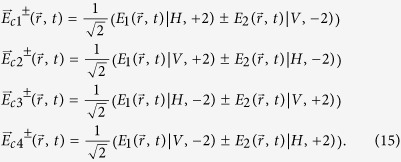


These manipulations, which result in eight distinguishable messages corresponding to Eq. (15), can be completed in Bob’s encoding station.

The output beam from the Bob’s encoding station was then combined with the other beam at Alice’s analyzer to perform decoding. The process of decoding contains two stages. One is that the polarization-OAM analysis is implemented with an apparatus consisting of a 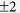-OAM splitter and a PBS on each path, as shown in Fig. 3(b). The splitter is composed of a binary forked grating with 30% diffraction efficiency into the first order and a pin-hole, which can transform an initial state with 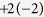-OAM into a Gaussian mode in the 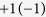 diffraction order^43^. Subsequently, the diffracting beams filtered by the pin-hole are merged by a PBS. The other is that the first-order correlations are measured. After the polarization-OAM analysis, the beam of port D is combined with the beam from port A and port B though a BS, respectively. Then each beam passes through the optical element group that includes BS, PBS@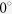, and PBS@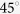, as shown in Fig. 3(c). We adjust appropriately the path length difference 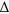 of the two beams, which corresponds to the phase 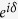 by the relation 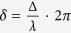. The distinguishable messages can be obtained directly by recording intensities 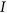 at eight detectors, which has been shown in Table 1 for theoretical results.

**Figure 4:**
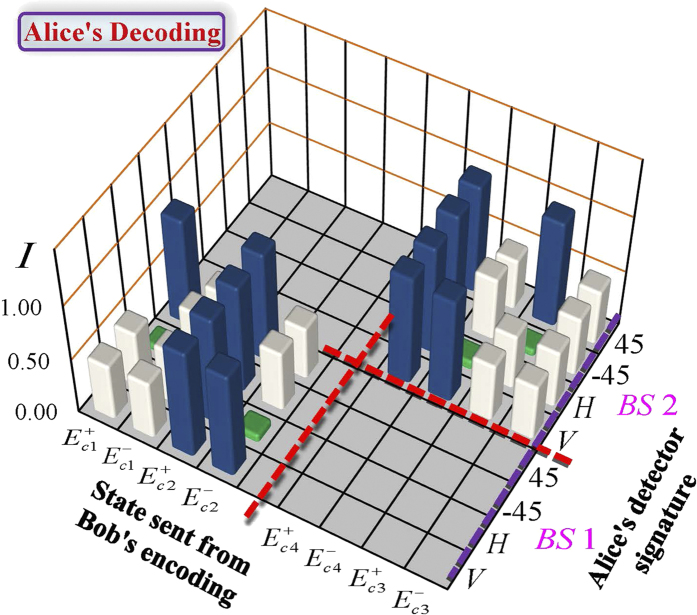
Experimental results of classical hypercorrelation-assisted dense coding. Normalization intensities were detected by Alice for each message sent by Bob. Three colors (blue, gray and green) of histogram represent the case of three-lever output.

In order to demonstrate the above theoretical analysis, we perform the corresponding experiment. The wavelengths of two independent light beams (produced by two independent He-Ne lasers) are still taken as 632.8nm. To characterize the observable intensity at Alice’s analyzer, we change the path length difference related to the phase. The results of experimental measurements are shown in Fig. 4. Three colors (blue, gray and green) of histogram represent the case of three-lever intensity output. Here the experimental data are normalized by the average value from all measured dates of detectors under maintaining the stability of interferometer for 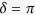. Such normalization is corresponding to the normalized condition 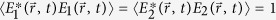. From the experimental results in Fig. 4, we can determine the message that Bob has sent according to the intensity difference of detectors. Therefore, Alice can distinguish completely the messages sent by Bob, implementing the information transfer of 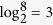 bits. Such high information transfer originates from the classical hypercorrelation as described in the above part.

If we do not use the classical hypercorrelation, consider 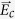 or 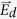 channel separately, the unified operation is used to encode messages in two DOFs, the maximum channel capacity is 2 bits (demonstration has been given in Section III of Supplemental Materials). This means that the present work provides the first demonstration that the superdense coding can be realized in the classical optics, which is analogy of quantum superdense coding using pairs of photons simultaneously entangled in spin and OAM as described in refs 13 and 14. 

Comparing the present classical scheme with those for quantum superdense coding described in ref. 13, we find that present scheme posses many advantages. First, the attainable channel capacity in the experiment for such a superdense coding can reach 3 bits, which is higher than that (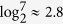) of usual quantum superdense coding using pairs of photons simultaneously entangled in spin and OAM. Second, it is very convenient to realize in optical communication, because the distinguishable messages can be directly identified from the detectors instead of interference and coincidence measurements.

In addition, we would like to point out that the present method is different from the signal demodulation in coherent optical communication although correlation properties of the classical optics have been used in the process^46^,^47^,^48^. The traditional coherent demodulation is based on polarization-division multiplexing (PDM) to increase the capacity of optical communication systems. For example, in ref. 46 the information is encoded onto the electrical field and modulated in orthogonal polarizations. In fact, in the present work we have presented a new method to improve the information capacity, which is based on the classical hypercorrelation corresponding to the quantum superdense coding using pairs of photons simultaneously entangled in the polarization and OAM. Such a method does not require the measurement of intensity difference of two outputs from BS 1 (BS 2), because it is enough to distinguish messages only by manipulating and measuring the optical intensity at one arm after the BS 1 (BS 2).

## Conclusions

In summary, we have demonstrated experimentally the classical hypercorrelation by implementing the measurement of CHSH inequality in every DOF: polarization and OAM. Such a classical hypercorrelation is similar to the production of hyper-entangled photon pairs from spontaneous down-conversion of nonlinear crystal. They can also be used to increase the information capacity of the system. We have realized experimentally the analogy of quantum superdense coding in the classical optics by using such classical hypercorrelation. Comparing the superdense coding in the quantum case using pairs of photons simultaneously entangled in polarization and OAM, it exhibits many advantages, for example, the attainable channel capacity in the present experiment can reach 3 bits, which is higher than that (2.8 bits) of usual quantum one. It is very convenient to realize in the classical optics, because the distinguishable messages can be directly identified from the detectors instead of interference and coincidence measurements. Thus, our study opens a new way to improve the information capacity in the classical optical communication. It not only provokes deep thought on some basic physical problems such as essence of entanglement and correlation, but also shows potential application in classical optical information processes.

## Additional Information

**How to cite this article**: Li, P. *et al.* Classical hypercorrelation and wave-optics analogy of quantum superdense coding. *Sci. Rep.* **5**, 18574; doi: 10.1038/srep18574 (2015).

## Supplementary Material

Supplementary Information

## Figures and Tables

**Table 1:** Overview of possible manipulations and theoretical results for the scheme in Fig. 3.

Bob’s encoding	Down channel		Up channel		BS 1				BS 2			
	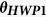	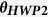	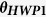	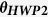	H	V	45	−45	H	V	45	−45
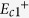	0	0			1	1	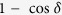	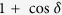				
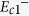	0				1	1	1	1				
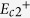	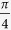				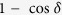	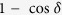	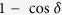	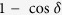				
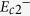	0	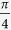			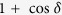	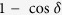	1	1				
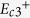			0	0					1	1	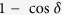	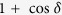
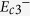			0						1	1	1	1
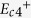			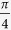						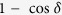	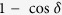	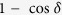	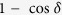
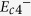			0	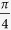					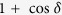	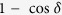	1	1

Abbreviation: BS, beam-splitter; HWP: half-wave plate.
