# Supplementary material for: Classical hypercorrelation and wave-optics analogy of quantum superdense coding
Source: Sci Rep. 2015 Dec 22;5:18574. doi: 10.1038/srep18574 (PMC4686973; doi:10.1038/srep18574)
Supplement: Supplementary Information [file srep18574-s1.pdf]

Supplementary Information

Classical hypercorrelation and wave-optics analogy of quantum superdense coding

Pengyun Li, Yifan Sun, Zhenwei Yang, Xinbing Song, and Xiangdong Zhang*

School of Physics, Beijing Institute of Technology and Beijing Key Laboratory of Fractional Signals and Systems, 100081, Beijing, China

I. Experimental demonstration of classical optical correlation in polarization degree of freedom (DOF)

In this section, we present the experimental demonstration of the classical optical correlation in polarization degree of freedom in detail. First, we can use Jones matrix $R_{\theta}(\frac{\theta}{2})=\begin{bmatrix}\cos(\theta) & \sin(\theta)\\ \sin(\theta) & -\cos(\theta)\end{bmatrix}$ to describe the rotation function of HWPs, where $\frac{\theta}{2}$ denotes fast axes at $\frac{\theta}{2}$. As is shown in Fig. 1(c), when the HWPs are rotated with the angle of $\frac{\theta_A}{2}$ and $\frac{\theta_B}{2}$, respectively. E_{a2} and E_{b2} are transformed to $\vec{E}_{a2}(\vec{r},t)=E_{a2h}(\vec{r},t)H+E_{a2v}(\vec{r},t)V$ and $\vec{E}_{b2}(\vec{r},t)=E_{b2h}(\vec{r},t)H+E_{b2v}(\vec{r},t)V$, here

$$
\begin{aligned}
E_{a2h}(\vec{r},t) &= \frac{1}{\sqrt{2}}\left(\frac{1}{2}E_1(\vec{r},t)\cos\theta_A+\frac{1}{2}E_2(\vec{r},t)\sin\theta_A\right)\\
E_{a2v}(\vec{r},t) &= \frac{1}{\sqrt{2}}\left(\frac{1}{2}E_1(\vec{r},t)\sin\theta_A-\frac{1}{2}E_2(\vec{r},t)\cos\theta_A\right)\\
E_{b2h}(\vec{r},t) &= \frac{1}{\sqrt{2}}\left(\frac{1}{2}E_1(\vec{r},t)\sin\theta_B+\frac{1}{2}E_2(\vec{r},t)\cos\theta_B\right)\\
E_{b2v}(\vec{r},t) &= \frac{1}{\sqrt{2}}\left(-\frac{1}{2}E_1(\vec{r},t)\cos\theta_B+\frac{1}{2}E_2(\vec{r},t)\sin\theta_B\right)
\end{aligned}
\quad . \qquad \text{(S1)}
$$

The correlation function is defined in the following form: [17]

$$C(\theta_A,\theta_B)=P_{hh}(\theta_A,\theta_B)-P_{hv}(\theta_A,\theta_B)-P_{vh}(\theta_A,\theta_B)+P_{vv}(\theta_A,\theta_B)\,. \qquad \text{(S2)}$$

where

$$P_{i,j}(\theta_A,\theta_B)=\frac{\left|\left\langle E_{a2i}{}^{*}(\vec{r},t)E_{b2j}(\vec{r},t)\right\rangle\right|^2}{I_0}$$
$$I_0=\sum_{i,j}\left|\left\langle E_{a2i}{}^{*}(\vec{r},t)E_{b2j}(\vec{r},t)\right\rangle\right|^2, \qquad i,j=h,v \qquad \text{(S3)}$$

In the experiment, the fields of output lights are not directly measured. However, the first-order field correlation can be obtained through measuring the difference of light intensities at two export positions on MZ interferometer, because $\left\langle E_{ai}(\vec{r},t)E_{bj}^{*}(\vec{r},t)\right\rangle \propto I_1-I_2=\Delta I$ ($i,j=h,v$). Here I_1 and I_2 represent the light intensities at two export positions of the MZ interferometer and ΔI is the difference between them. More information about the measurement method for the first-order field correlation is provided in Ref. 27. Then, the CHSH measurement is given by

$$S=\left|C(\theta_A,\theta_B)+C(\theta_A,\theta_B{}')+C(\theta_A{}',\theta_B)-C(\theta_A{}',\theta_B{}')\right|. \qquad \text{(S4)}$$

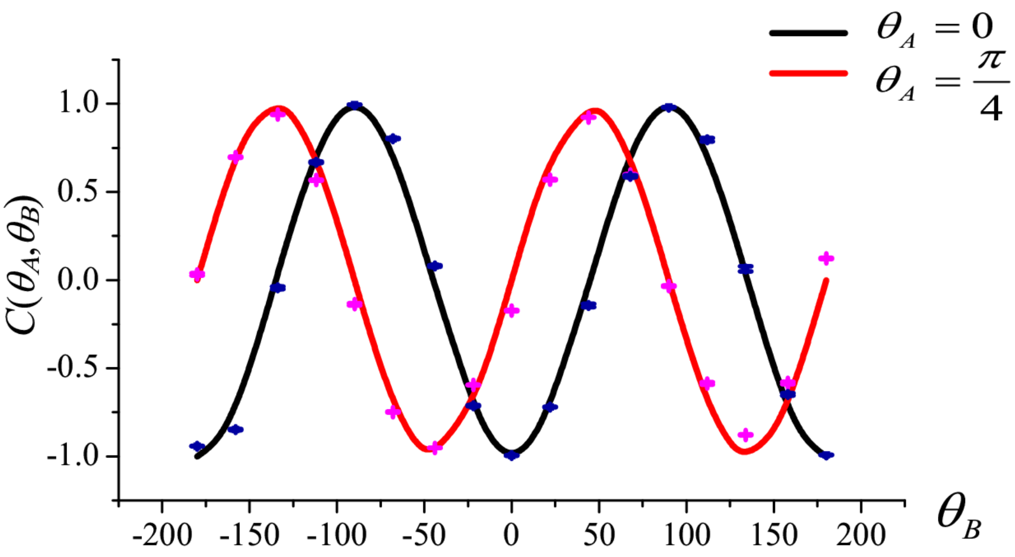

Figure S1. Correlation functions as a function of polarization rotated angle θ_B at $\theta_A=0$ and $\theta_A=\frac{\pi}{4}$, respectively. The solid lines and dots (square and triangular) represent theoretical and experimental results, respectively.

Figure S1 shows experimental results for the normalized correlation functions $C(\theta_a,\theta_b)$ as a function of polarization rotated angle θ_B at $\theta_A=0$ and $\theta_A=\frac{\pi}{4}$, respectively. Based on experimental results and Eq. (S4), we can obtain $|S_{Pol}|_{\max}=2.579\pm0.012$ as $\theta_A=0,\theta_A'=\frac{\pi}{4},\theta_B=-\frac{\pi}{8}$ and $\theta_B'=\frac{\pi}{8}$. Contrary to commonly-perceived local realism theories, the

CHSH inequality is violated apparently, which can provide a demonstration of polarization correlation.

II. The re-construction of classical hyper-correlated state

In this section, we present the experimental demonstration for the classical hypercorrelation, which is generated from the simplified correlation source described in Fig. 3(a), from observing a Bell-type inequality violation in each DOF. The output fields from the correlation source described in Fig. S2(a) have been given in Eq. (14). The scheme consists of two parts: the source generating classical hypercorrelation states (Fig. S2(a)), and the measurement insets for demonstrating the correlation properties (Fig. S2(b), (c) and (d)). The source and the method of measurement shown in Fig. S2(a) and Fig. S2(d) are agreement with those shown in Fig. 3(a) and Fig. 1(d). The measurement setups shown in Fig. S2(b) and Fig. S2(c) have some differences compared with those shown in Fig. 1(b) and Fig. 1(c). Comparing with Fig. 1(b), in Fig. S2(b) we have added a new interferometer (including a PBS， a BS and a HWP) to erase polarization labels. Similarly, we have added a new interferometer (including a PBS， a BS and a Dove prism) to erase OAM labels in Fig. S2(c) comparing with Fig. 1(c).

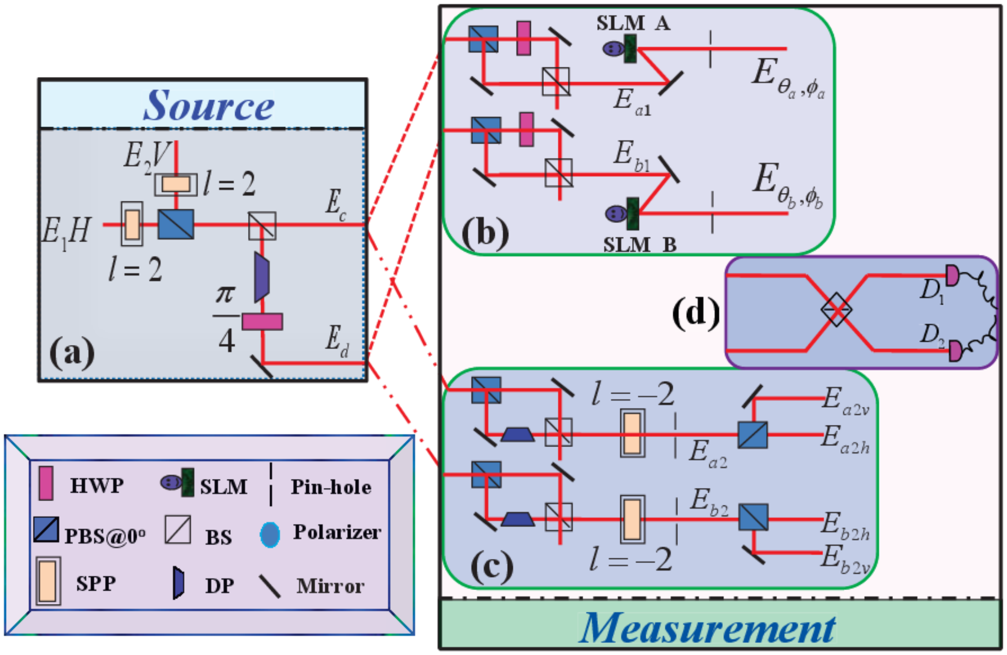

Figure S2. Experimental setup for hyper-correlated demonstration. (a) The preparation of hyper-correlated source; (b) and (c) show the demonstration of OAM correlation and polarization correlation, respectively; (d) The measurement is performed by the first-order correlation.

After the beams from $\vec{E}_c(\vec{r},t)$ and $\vec{E}_d(\vec{r},t)$ pass through the experimental setup in Fig.

S2(b), they can be transformed into

$$\begin{aligned}\vec{E}_c'(\vec{r},t) &= \frac{1}{\sqrt{2}}\left(\frac{1}{\sqrt{2}}E_1(\vec{r},t)\,|+2) + \frac{1}{\sqrt{2}}E_2(\vec{r},t)\,|-2)\right)\otimes|V) \\ \vec{E}_d'(\vec{r},t) &= \frac{1}{\sqrt{2}}\left(\frac{1}{\sqrt{2}}E_1(\vec{r},t)\,|-2) + \frac{1}{\sqrt{2}}E_2(\vec{r},t)\,|+2)\right)\otimes|V)\end{aligned}. \qquad \text{(S5)}$$

Similarly, the beams go through the optical element group shown in Fig. S2(c), the fields are described as

$$\begin{aligned}\vec{E}_c''(\vec{r},t) &= \frac{1}{\sqrt{2}}\left(\frac{1}{\sqrt{2}}E_1(\vec{r},t)\,|H) + \frac{1}{\sqrt{2}}E_2(\vec{r},t)\,|V)\right)\otimes|+l) \\ \vec{E}_d''(\vec{r},t) &= \frac{1}{\sqrt{2}}\left(\frac{1}{\sqrt{2}}E_1(\vec{r},t)\,|V) + \frac{1}{\sqrt{2}}E_2(\vec{r},t)\,|H)\right)\otimes|+l)\end{aligned}. \qquad \text{(S6)}$$

Using a SPP with $l=-2$ and a pin-hole in each path, Eq. (S6) becomes

$$\begin{aligned}\vec{E}_c'''(\vec{r},t) &= \frac{1}{\sqrt{2}}\left(\frac{1}{\sqrt{2}}E_1(\vec{r},t)\,|H) + \frac{1}{\sqrt{2}}E_2(\vec{r},t)\,|V)\right)\otimes|0) \\ \vec{E}_d'''(\vec{r},t) &= \frac{1}{\sqrt{2}}\left(\frac{1}{\sqrt{2}}E_1(\vec{r},t)\,|V) + \frac{1}{\sqrt{2}}E_2(\vec{r},t)\,|H)\right)\otimes|0)\end{aligned}. \qquad \text{(S7)}$$

By using normalized condition $\left\langle \frac{1}{\sqrt{2}}E_1^*(\vec{r},t)\cdot\frac{1}{\sqrt{2}}E_1(\vec{r},t)\right\rangle = \left\langle \frac{1}{\sqrt{2}}E_2^*(\vec{r},t)\cdot\frac{1}{\sqrt{2}}E_2(\vec{r},t)\right\rangle = 1$, we find that Eq. (S5) is identical with Eqs. (4) and (5), Eq. (S7) is also identical with Eqs. (11) and (12). Thus, the violation of CHSH Bell inequality for the OAM and polarization DOF can be verified in such a case according to the method described in the text.

III. The channel capacity without hypercorrelation

In this section, we present the calculation results on the channel capacity without hypercorrelation. That is, Bob encodes messages in two DOFs by operating globally the beam E_c, and then Alice decodes the messages directly. Because the beam E_c is composed of two completely incoherent beams E_1 and E_2, the relative phase between E_1 and E_2 is completely meaningless. When performing unified operations for two independent DOFs, Bob can encode his messages in polarization DOF in the following form:

$$\begin{cases} |H)_1 + |V)_2 \\ |V)_1 + |H)_2 \end{cases} \qquad \text{(S8a)}$$

or

$$\begin{cases} |H)_1 + |H)_2\,, \\ |V)_1 + |V)_2 \end{cases} \qquad \text{(S8b)}$$

where the subscripts 1 and 2 indicate the beam E_1 and E_2, respectively. Similarly, in the OAM

DOF, the messages can be expressed as

$$\begin{cases} |+2\rangle_1 + |-2\rangle_2 \\ |-2\rangle_1 + |+2\rangle_2 \end{cases} \qquad \text{(S9a)}$$

or

$$\begin{cases} |+2\rangle_1 + |+2\rangle_2 \\ |-2\rangle_1 + |-2\rangle_2 \end{cases}. \qquad \text{(S9b)}$$

Obviously, for the E_c, Bob can encode information by performing one of 2×2 possible operations. For two groups of possible information in Eqs. (S8b) and (S9b), they can be distinguished easily by using one PBS and a SPP with $l=2$. However, two groups of possible information in Eqs. (S8a) and (S9a) can not be distinguishedin in the present case due to the complete incoherent condition between E_1 and E_2. This means that Alice can only get four possible messages in the channel. According to the information theory[49], the channel capacity is defined as

$$\begin{aligned} C &= R_{\max} = \max\left[H(X) - H(X/Y)\right] \\ H(X) &= -\sum_{i=1}^{N} p(x_i)\log_2 p(x_i) \end{aligned}, \qquad \text{(S10)}$$

where R represents the information transmission rate, $X\,(Y)$ denotes the symbol for the input (output) channel, $H(X)$ is a function of $p(x_i)$ and represents Shannon entropy, $p(x_i)$ is the probability of event x_i. The ideal channel capacity C can be obtained when R reaches to maximum and $p(x_i)=\frac{1}{N}$. Thus, in this scheme, the maximum channel capacity can be given by $C=-\sum_{i=1}^{4} p(x_i)\log_2 p(x_i) = -4\times\frac{1}{4}\log_2\frac{1}{4} = 2$ bits.
